# Application of Psychometric Methods in Dimensional Analysis and Integration of Assessment Tools in Early Diagnosis for Autism Spectrum Disorder

**DOI:** 10.1002/jclp.70059

**Published:** 2025-10-26

**Authors:** Ilenia Le Donne, Monica Mazza, Margherita Attanasio, Nicole Covone, Maria Paola Greco, Veronica Scurti, Marco Valenti

**Affiliations:** ^1^ Department of Biotechnological and Applied Clinical Sciences University of L'Aquila Italy; ^2^ Reference Regional Centre for Autism, Abruzzo Region, Local Health Unit ASL1 Italy; ^3^ Department of Health Abruzzo Region Italy

**Keywords:** ADOS‐2, assessment, autism, exploratory graph analysis, psychometry, structural equation model

## Abstract

**Background:**

Early diagnosis of autism spectrum disorder (ASD) is crucial for timely intervention, and requires reliable and valid screening and diagnostic tools. The Toddler Module of the Autism Diagnostic Observation Schedule, Second Edition (ADOS‐2), is widely used but its factor structure and applicability in the clinical practice need further investigation.

**Objective:**

This study aimed to explore the underlying dimensional structure of the ADOS‐2 Toddler Module in Italian context, and to examine the association between the identified ASD symptom dimensions and cognitive development, as measured by the Griffiths Mental Development Scales (GMDS).

**Methods:**

The study was conducted in two phases: in Phase 1, Exploratory Graph Analysis (EGA) was used to identify latent dimensions of the ADOS‐2 Toddler Module items in a sample of 91 Italian children aged 12–30 months at risk for ASD. In Phase 2, structural equation modeling (SEM) was performed on a subsample of 60 children who completed the GMDS to examine associations between ASD symptom dimensions and cognitive development.

**Results:**

EGA revealed a stable three‐factor structure underlying the Toddler Module items. The SEM analysis demonstrated a good model fit and significant associations between ASD symptom dimensions and the Personal‐Social subscale of the GMDS.

**Conclusions:**

These findings provide preliminary evidence for a three‐dimensional structure of ASD symptoms in very young children and suggest that cognitive development, particularly social‐personal skills, is related to early ASD symptomatology. The results have potential implications for refining early diagnostic assessments and guiding clinical practice.

## 1 Introduction

Early diagnosis is crucial in improving the prospects for intervention and support for children with autism spectrum disorder (ASD), enabling the timely implementation of individualized educational and therapeutic strategies. However, the variability of clinical manifestations in ASD makes early diagnosis a significant challenge for clinicians and researchers. ASD is a neurodevelopmental condition characterized by difficulties in social interaction and communication and repetitive behaviors and restricted interests (American Psychiatric Association, D.S.M.T.F., & American Psychiatric Association, D. S 2013; Lai et al. 2014). This symptomatology typically emerges at an early age and can be identified around 2 years, though this age varies in regressive and “plateau” forms of ASD (Ozonoff and Iosif 2019; Pino et al. 2024). There is a substantial body of literature on the diagnosis of ASD, which involves gathering information from parents or caregivers, observing the child, and administering validated diagnostic instruments. In this context, the Autism Diagnostic Observation Schedule ‐ Second edition (ADOS‐2) (Lord et al. 2012) is widely used as a tool for diagnosing ASD. The ADOS‐2 is a semi‐structured, standardised observational tool that provides a quantifiable picture of ASD‐associated characteristics. This tool has been translated and edited into several languages including Italian, although the data for validation of the tool currently in use refer to a US sample. It consists of five modules that can be administered based on the subject's chronological age and expressive language level, including the Toddler Module aimed at children aged 12 to 30 months (Lord et al. 2012). Several studies have investigated the sensitivity and specificity diagnostic algorithms, and dimensions of ADOS‐2, particularly for Modules 1, 2, 3, and 4 (de Bildt et al. 2011; Hong et al. 2021; Kamp‐Becker et al. 2013; Medda et al. 2019). For the Toddler Module, however, studies on its use in clinical practice remain limited (Hong et al. 2021; Lee et al. 2019). This Module through cut‐off scores, establishes levels of risk for receiving an autism diagnosis, acknowledging diagnostic uncertainty due to developmental variability or confounding conditions often present in younger children (Esler et al. 2015). A confirmed diagnosis allows for immediate, individualised rehabilitation pathways. In this framework, the application of psychometric methods in clinical practice is also essential, where optimising information collection and reducing errors by eliminating uninformative items are key to maintaining accuracy (Donadello et al. 2017). This approach has been explored by applying Formal Psychological Assessment (FPA) to all ADOS‐2 Modules (Pino et al. 2018) investigating relationships between items and clinical observations to improve data quality and enable adaptive assessment for individuals with ASD. This has particular relevance in clinical practice, especially when assessing children as young as 12 months who present with suspected ASD and undergo a demanding assessment requiring sustained collaboration.

An additional factor to consider is that some behavioural and clinical manifestations typical of autism overlap with other neurodevelopmental conditions, such as developmental delay (Veness et al. 2014; Ventola et al. 2007). Studies on the developmental trajectories and symptomatic manifestation of ASD (Landa et al. 2007; Ozonoff et al. 2010; Ozonoff and Iosif 2019; Pino et al. 2024) have highlighted how social communication and behavioral patterns in children later diagnosed with ASD may vary within the second year of life. Therefore, assessing developmental indicators for early identification of at‐risk children is crucial in clinical practice. The support of tools assessing these indicators is vital for gathering information that strengthens confidence in an early diagnosis. Numerous studies indicate that understanding early ASD onset is linked to social behaviour indicators, such as social interest and shared affection, which appear within the first 2 years of life (Ozonoff and Iosif 2019). It has also been shown that some developmental skills measured by a cognitive development scale, such as the Griffiths Mental Development Scales (GMDS), affect symptom severity at the time of diagnosis (Pino et al. 2024).

However, although these instruments are widely used in clinical practice, their psychometric validation within the Italian context remains limited, particularly for the ADOS‐2 Toddler Module, for which cultural adaptation has not yet been supported by systematic validation studies. This limitation highlights the importance of applying advanced psychometric analyses to examine the dimensional structure of the ADOS‐2 and its relationship with developmental indicators such as those assessed by the GMDS in Italian clinical populations. Thus, one of researchers’ primary goals is to highlight the importance of multidisciplinary approaches, such as the combined use of diagnostic tools and innovative analytical methods, to better standardise and understand the manifestations of ASD symptoms.

Recently, psychometric networks using network models to represent complex relationships between variables have been applied in psychological research. One such method is explorative graph analysis (EGA) (Golino and Epskamp 2017). EGA enables the identification of dimensions within multivariate data by employing network models, where nodes (circles) represent variables and edges (lines) represent relationships (e.g., partial correlations) between pairs of nodes. Clusters or relationships between nodes reveal the network's dimensions (Christensen and Golino 2021). Based on these premises, this study was designed as an integrated analysis conducted in two phases. In Phase 1, we aimed to replicate and investigate the underlying structure of the Toddler Module items in an Italian population of children aged 12 to 30 months, with the goal of identifying latent factors and any patterns or clusters of related items. By analysing the connection patterns between items, we can assess the internal consistency of the Toddler Module and identify any items that may not align with the test's overall structure. Additionally, we examined the structured tasks of the Toddler Module that support the coding of items selected for the diagnostic algorithm, investigating the primary dimensions that explain ASD symptomatology in very young children. This approach enables us to differentiate essential items from redundant ones, facilitating an adaptive and efficient assessment process for very young children.

In Phase 2, we investigated the relationship between the constructs identified in the Phase 1 and those assessed by the GMDS (see Measures section) using structural equation modelling (SEM). This theoretical model provides a framework for exploring how cognitive development (as measured by the GMDS) may influence the symptom dimensions measured by the Toddler Module, how various early symptoms of autism manifest and interact, and how integrating different measures can support the investigation of core aspects of the condition.

This approach would supports clinical practice by facilitating the collection of key information, allowing clinicians to focus on essential features for identifying at‐risk children. It enables a more adaptive assessment of child characteristics while also reducing the time required to administer diagnostic tools, thereby supporting timely and targeted intervention planning.

## 2 Methods

### 2.1 Participants

The study comprised two analytical phases.

The Phase 1 involved a clinical sample of 91 children with an age range of 12 to 31 months (mean age in months = 24.94 ± 4.25) at first clinical consultation who arrived for suspected ASD at the […], Italy.

For the second phase of the study, a subsample of 60 children from the original cohort (*N* = 91) also completed a functional developmental assessment using the Griffiths Mental Development Scales (GMDS) alongside the ASD symptom assessment.

Table 1 summarizes the demographic and clinical characteristics of the full sample and the subsample.

**Table 1:** Demographic and clinical characteristics of the sample and the GMDS subsample.

	Full Sample (Phase 1)	GMDS Subsample (Phase 2)
	*N* = 91	*N* = 60
*Gender (n‐%)*		
Male	63 (69.2%)	41 (68.3%)
Female	28 (30.8%)	19 (31.7%)
*Mean age in months (SD)*	24.79 (4.6)	25.07 (4.12)
*ADOS‐2, Module Toddler*		
AS total	15.58 (4.3)	15.91 (4.6)
CRR total	3.59 (1.9)	3.51 (2)
Total	19.21 (5.45)	19.46 (5.7)
*Level of risk (n‐%)*		
None to mild	3 (3.3%)	3 (5%)
Mild to moderate	11 (12.1%)	5 (8.3%)
Moderate to severe	77 (84.6%)	52 (86.7%)
*GMDS*		
*Development quotient*	—	78.9 (16.8)
*Development age*	—	19.94 (5.15)

### 2.2 Procedures

Children underwent a neuropsychiatric evaluation using standardized measures and clinical observation in accordance with the criteria of the Diagnostic and Statistical Manual of Mental Disorders (5th ed.; DSM‐5) (American Psychiatric Association, D.S.M.T.F., & American Psychiatric Association, D. S 2013) performed by a multidisciplinary team experienced in the diagnosis of ASD. Following the initial neuropsychiatric examination, the children underwent an assessment of ASD symptomatology (please refer to the “measures” section) conducted by a trained clinical psychologist.

The study was conducted in accordance with the Declaration of Helsinki and good clinical practice guidelines. Ethical approval was obtained from the Ethics Committee of the NHS Local Health Authority […]. Written informed consent was obtained from all parents or legal guardians, who also completed a sociodemographic questionnaire before test administration.

### 2.3 Measure

#### 2.3.1 Autism Diagnostic Observation Schedule‐ Second Edition, Module Toddler

ADOS‐2 (Lord et al. 2012) is a semi‐structured observational instrument, considered as a gold‐standard measure in clinical diagnosis for ASD and, often, required for access to early interventions. The edition translated into Italian by Colombi et al. (2013) was used in the present study. It consists of 5 modules that are selected and administered based on the level of expressive language and the chronological age of the subject. Each Module consists of several tasks that elicit autism‐related behaviors, requiring between 40 and 60 min of administration time. Each Module, except Module 4, provides a total score and scores for the two domains of Social Affect (SA) and Restricted and Repetitive Behaviors (RRB). Comparison scores for symptom severity are, in addition, provided. Only the Toddler Module was considered in the study. This Module allows assessment of early autism‐related symptoms in children 12 to 30 months of age, providing a range of risks. There are 11 tests, plus 4 additional ones, which allow coding of the items selected to represent the two diagnostic algorithms: 1) “All younger/older with few or no words” algorithm, and 2) “Older with some words” algorithm.

Regarding the present study, our initial sample consisted of 108 children; however, only 17 children were the “Older with some words” algorithm coded. Thus, our focus was on the performance of the 14 items of the “All younger/older with few or no words” algorithm.

#### 2.3.2 Griffith Mental Developmental Scale

The GMDS (Griffiths 1954, 1970) allows the assessment of cognitive development in children aged 0‐8 years through two scales: the 0–2 scale and the 2–8 years scale. The GMDS‐R 0–2 years allows assessment of development in children up to 24 months of age, while the 2‐8 scale represents the revised and extended version (GMDS‐ER) dedicated to children up to 8 years of age (Griffiths and Huntley 1996; Luiz et al. 2006).

Translated and edited versions in Italian were used in the present study (GMDS 0‐2: Battaglia and Savoini 2007; GMDS‐ER: Sannio Fancello et al. 2007).

The scales contain 6 subscales: Subscale A, Locomotor, for postural development, walking and motor skills; Subscale B, Personal‐Social, which covers social interaction skills and adapting to the environment; Subscale C, Hearing and Language, assessing the main stages of language and communication development and assesses attention to sounds, vocalization production and early lexical development; the D subscale, Eye‐Hand Coordination, which assesses visual control and fine‐motor skills; the E subscale, Performance, which allows assessment of visual perception, speed of work and accuracy. Beginning at age 2 and thus present in the GMDS‐ER scale is subscale F, Practical Reasoning, consisting of items that assess the ability to use knowledge learned from the environment to solve problems and to understand mathematical concepts and moral problems.

Previous studies (Pino et al. 2024) have shown that two subscales are influential for ASD symptom severity, so for our theoretical model, we considered the two subscales of the GMDS, the Personal‐Social and Hearing‐Language (Scl and Lng).

### 2.4 Statistical Analysis

#### 2.4.1 Descriptive Analysis

Descriptive analysis were conducted on the samples (Table 1), using IBM SPSS (Version 27.0) (Crop 2020).

#### 2.4.2 Phase 1: Exploratory Graph Analysis

R software (Team 2021)(Team, R. C 2023), and the R Studio environment (Team, RStudio 2023) was used to conduct EGA. In particular, the following packages in R software were used: EGAnet (Golino and Christensen 2023), bootnet (Epskamp et al. 2018), qgraph (Epskamp et al. 2012), psych (Revelle and Revelle 2015). At this stage, the analyses were conducted on the entire sample of 91 children who were administered the ADOS‐2 Toddler Module.

Before performing the EGA (Golino and Epskamp 2017), a preliminary item analysis was conducted using the ‘All the smallest/the largest with few or no words’ algorithm to assess the normality of items and identify any excessive correlations (rho > 0.85) between them (Tabachnick and Fidell 2007).

The EGA approach was then applied to assess the underlying structure of the Toddler Module items, aiming to identify latent factors and any clusters of items that are correlated with each other. The main purpose of EGA is to detect clusters of highly connected nodes (or communities) based on functional separation (modularity) (Newman 2006), which indicates whether each function or dimension assesses a distinct feature of the evaluated construct. The GLASSO method with polychoric correlations was used to estimate the number of factors or dimensions (Epskamp and Fried 2018; Golino and Epskamp 2017). The result is a sparse network displaying nodes (items) and their relationships as lines (correlations). The exact number of dimensions was further examined using the Walktrap algorithm (Golino and Epskamp 2017; Pons and Latapy 2005). To assess the generalisability of the results, we evaluated the stability of the dimensions and items using a bootstrap approach (Christensen and Golino 2021). A parametric bootstrap with 10,000 iterations was performed, yielding descriptive statistics such as the median, 95% confidence intervals, and frequency of factor numbers. Stability assessment involves evaluating the frequency with which dimensions are replicated and how consistently each item appears within a specific dimension (Christensen and Golino 2021). According to Christensen and Golino (2021), item stability ranges from 0 to 1, with values of ≥ 0.70 being considered acceptable, suggesting that an item is stable and consistently present within a dimension.

In addition, network loading (i.e., standardized node strength) is used to evaluate the contribution of each item (or node) for dimension consistency. The magnitudes of the loadings were interpreted as follows: small: λEGA > 0.15; moderate: λEGA > 0.25; large: λEGA > 0.35 (Christensen and Golino 2021; H. Golino et al. 2020). Finally, a CFA was applied and goodness of fit was evaluated by calculating the comparative fit index (CFI), root mean square of error approximation (RMSEA), the goodness of fit index (GFI), and Chi‐square/degrees of freedom (Chisq/df) where a good fit was considered by CFI > 0.90; RMSEA < 0.08, GFI > 0.90 and Chisq/df < 3.

#### 2.4.3 Toddler Module Task and Items

Next, we identified the items assessed through the structured tasks of the Toddler Module, based on the ADOS‐2 manual, and determined which of these correspond to the symptom dimensions of ASD identified by the EGA analysis. This approach supports a more adaptive assessment, whereby redundant tasks can be administered only when necessary. The results of the qualitative analysis are provided in the Supporting Material (Table SM1).

#### 2.4.4 Phase 2: Structural Equation Modeling

The following R packages were used: psych (Revelle and Revelle 2015), dplyr (Wickham et al. 2023), ggplot2 (Wickham 2016), lavaan (Rosseel 2012), semTools (Jorgensen et al. 2025), semPlot (Epskamp 2015).

Structural Equation Modeling (SEM) was applied to the subsample of 60 children who had complete scores on both the ADOS‐2 and GMDS subscales.

We preliminarily examined a theoretical model by applying SEM to investigate the relationships between the latent factors of ASD symptomatology identified in the previous analysis and two cognitive development subscales of the GMDS: the Personal‐Social scale and the Language scale (Scl and Lng). These subscales were selected based on prior studies indicating their predictive value for ASD symptomatology (Pino et al. 2024).

SEM represents a multivariate statistical analysis technique in which various theoretical models can be tested that hypothesize how sets of variables define constructs and how these constructs are related to each other (Schumacker and Lomax 2015; Stein et al. 2012). SEM involves estimating the parameters of a system of simultaneous equations and includes other techniques such as regression, factor analysis, and path analysis (Stein et al. 2012).

Based on the network‐derived factors from the first part of the study, we specified a model comprising three first‐order latent variables—Social Interaction (SI), Communication Behaviours (CB), and Sensory/Stereotyped Behaviours (SSB)—each defined by a subset of ADOS‐2 Toddler Module items. These factors were then modelled as indicators of a second‐order latent construct representing overall ASD symptomatology (ASD). The ASD construct was subsequently regressed on the GMDS Personal‐Social and Language scales to assess their potential predictive contribution.

The model was estimated using the robust maximum likelihood estimator (MLR), which provides standard errors and test statistics that are robust to non‐normality in the data. Latent correlations, factor loadings, regression coefficients, and R² values were freely estimated, and standardized estimates were reported.

The goodness of fit of the SEM to the data was evaluated according to guidelines as follows: a non‐statistically significant χ^2^, a CFI ≥ 0.95 for a good fit, RMSEA with a 90% confidence interval (CI) of RMSEA ≤ 0.08, an SRMR ≤ 0.08 (Brown 2015; Hoyle 2012; Kline 2018).

## 3 Results

### 3.1 Phase 1: Explorative Graph Analysis

Table 2 shows the descriptive statistics of the Toddler Module items. The items show a univariate normal distribution with skewness and kurtosis within the desired thresholds.

**Table 2:** Descriptive statistics of the Toddler Module items.

Item		Mean (SD)	Skewness	Kurtosis	SE
A2	Frequency of spontaneous vocalizations directed at others	2.36 (0.78)	−0.86	−0.41	0.08
A8	Gestures	1.69 (1.06)	−0.14	−1.27	0.11
B1	Unusual eye contact	1.89 (0.67)	−0.95	1.65	0.07
B4	Facial expressions directed to others	1.21 (0.85)	0.03	−0.92	0.09
B5	Integration of gaze and other behaviors during social initiations	1.85 (0.93)	−0.11	−1.18	0.10
B6	Shared enjoyment in interaction	1.65 (1.06)	−0.22	−1.19	0.11
B12	Showing	2.51 (0.91)	−1.67	1.50	0.10
B13	Spontaneous initiation of joint attention	2.12 (0.94)	−0.87	−0.17	0.10
B14	Joint attention response	1.67 (0.83)	0.09	−0.77	0.09
B15	Quality of social initiations	1.84 (0.91)	0.15	−1.47	0.10
A3	Intonation of vocalizations and verbalizations	0.65 (0.92)	1.07	−0.19	0.10
D1	Unusual sensory interest in play materials/people	1.01 (0.85)	0.52	−0.39	0.09
D2	Hand and finger movements/posture	0.87 (0.95)	0.57	−1.01	0.10
D5	Unusual repetitive interests or stereotypical behaviors	1.29 (0.85)	0.08	−0.72	0.09

**Figure 1:**
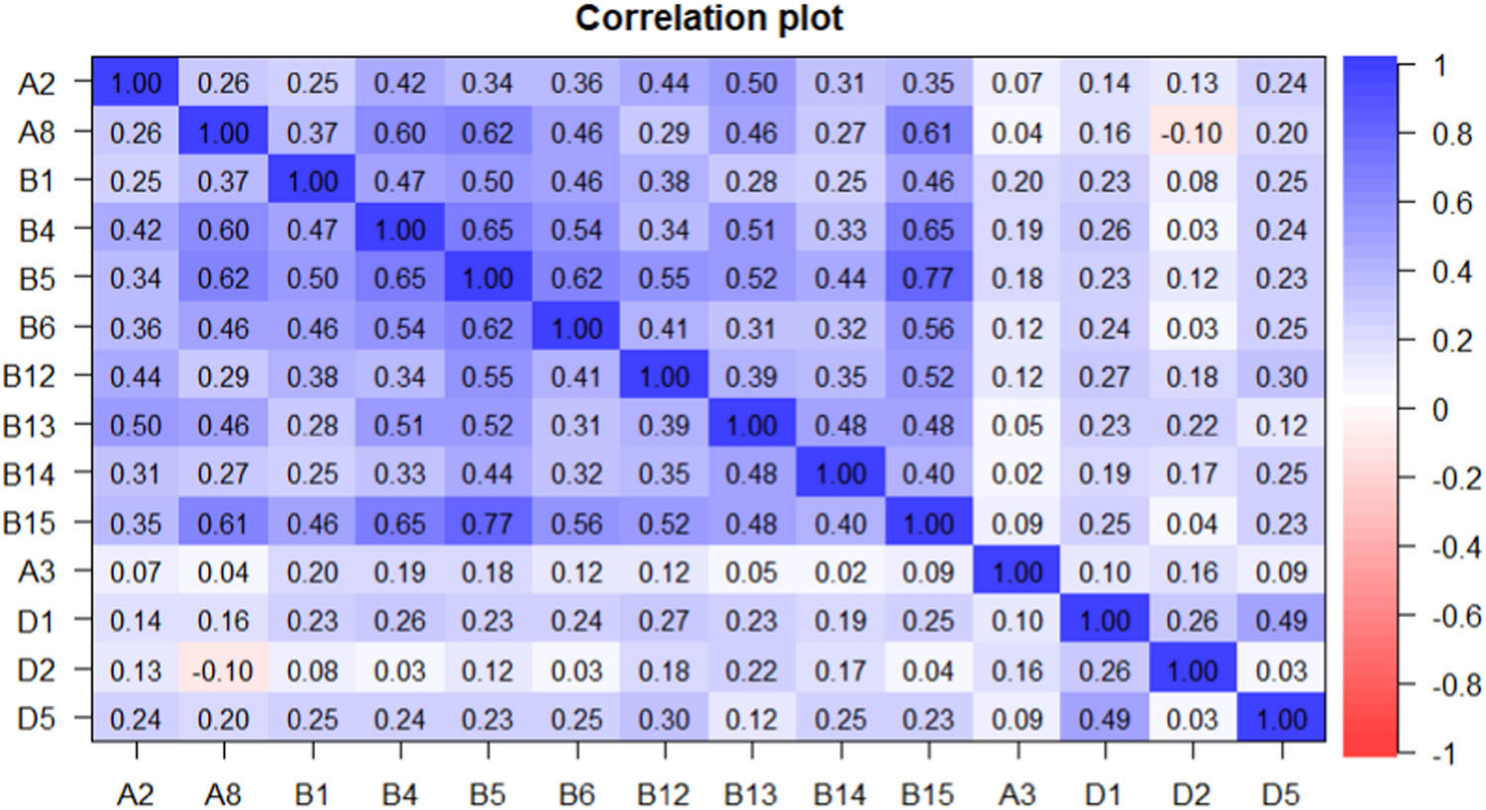
Heatmap of the Spearman's coefficients among Toddler items. Heatmap shows the association between the items, assessed with Spearman correlation coefficients. The figure shows low to moderate correlations and yet negative correlations between items A8 and D2 (r = −0.10).

The association between the items was assessed with Spearman correlation coefficients (Figure 1); the results show low to moderate correlations and yet negative correlations between items A8 and D2 (*r* = −0.10).

**Figure 2:**
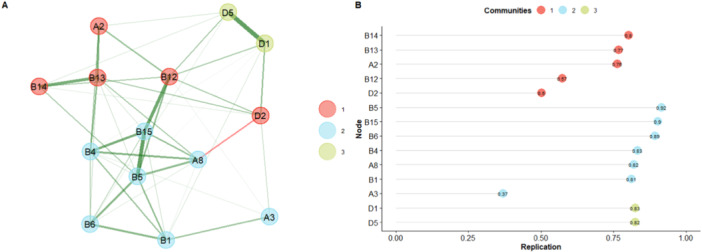
Graphical representation of the network and stability of items obtained from EGA. Exploratory graph analysis (A) and item stability (B) results from the Toddler Module items. (A) reports the first 3‐dimensional EGA model. The nodes represent the items of the Toddler Module and the edges (lines) their regularized partial correlations; the thickness of the edges represents strength of the correlation. Each dimension (communities) is represented with a different color. (B) reports the stability of the items within each dimension (communities); on the x‐axis are the values of the individual items (values > 0.70 were considered acceptable).

The EGA results show a network characterized by 3 dimensions (Figure 2A); the bootstrapped EGA results also show a 3‐dimensional solution (median = 3; 95% CI [1.41−4.58]). Furthermore, the analysis indicates that the 3‐dimensional solution characterizes the network by 46%, indicating low structural stability. Analysis of item stability within the dimensions found also shows the presence of unstable items (< 0.70) in dimensions 1 and 2 (Figure 2B), particularly items A3, B12, and D2.

To find a better structural solution, we re‐analyzed the network with the elimination of the three unstable items highlighted by the EGA.

**Figure 3:**
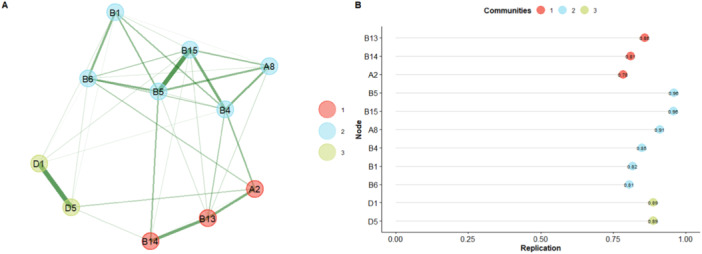
The second model obtained from EGA results after the elimination of inconsistent items. Second model of the exploratory graph analysis and item stability results. (A) reports the 3‐dimensional EGA model after deleting the unstable items obtained from the first model (A3, B12, D2). (B) reports the stability of the items within each dimension.

The results of the second EGA model (Figure 3A) show a 3‐dimensional solution again, confirmed by the bootstrapped EGA (median = 3; 95%CI [1.83–4.12]); the dimensions found were obtained for 69% of the simulated network. The structural consistency results show good stability for Dimension 1 (74%), Dimension 2 (67%), and Dimension 3 (88%). The good structural stability is also confirmed by analyzing the stability of items within dimensions. The results show that the items exhibit good stability (> 0.70) (Figure 3B).

Because the EGA grouped the items differently and identified 3 dimensions in the Toddler's structure than previous theorizing performed on the ADOS‐2 (Gotham et al. 2007), we performed a semantic content analysis of the grouped items. The review indicated that Dimension 1 items would assess behaviors related to “Social Intentionality,” Dimension 2 items would assess aspects related to “Communicative Behaviors,” and Dimension 3 items represent the area of “ Sensory and Stereotyped Behaviors.”

In addition, the analysis of network loadings (Table 3) showed whether each item has an association and is consistent with the dimension. Some items showed cross‐loadings, but these values have a range from zero to small, being lower than the loading on the assigned dimension. In the dimension “Social Intentionality,” λEGA ranged from 0.39 (large) to 0.17 (small); the dimension “Communicative Behaviors” showed a λEGA of 0.45 (large) to 0.24 (moderate); the dimension “Sensory and Stereotyped Behaviors” showed a λEGA of 0.39 (large).

**Table 3:** EGA‐based network loadings.

	Dimensions		
Item	Social intentionality	Comunication behaviors	Stereotyped and sensory behaviors
B13	0.374	0.154	0
B14	0.205	0	0
A2	0.177	0.108	0
B5	0.144	0.45	0
B15	0	0.441	0
B4	0.205	0.318	0
B6	0	0.262	0
B1	0	0.243	0
A8	0	0.24	0
D1	0	0	0.399
D5	0.123	0	0.399

As a confirmation of the structural model, it was further investigated by CFA. The model fitting data resulted in CFI = 0.95, RMSEA = 0.07, GFI = 0.97, and Chisq/df = 1.48.

### 3.2 Phase 2: Structural Equation Model

**Figure 4:**
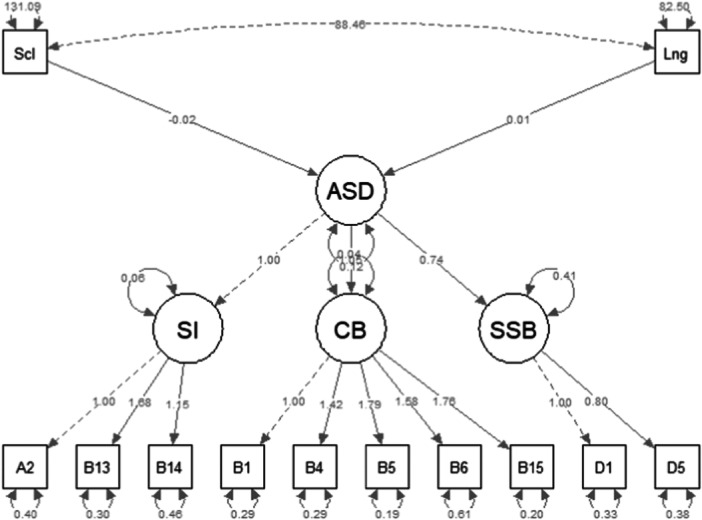
Graphical representation of the theoretical SEM model. Graphical representation of the theoretical SEM model for measuring the patterns of the Personal‐Social (Scl) and Language (Lng) Subscales of the GMDS and the latent factors of the Toddler Module that emerged in the first study, namely Social Intentionality (SI), Comunicative Behaviours (CB) and Sensory and Stereotyped Behaviors (SSB). Estimating the loadings of the examined items of the Toddler Module on the three latent factors reveals that they are associated with the general construct of ASD symptomatology. In addition, the two GMDS subscales and ASD symptomatology show a significant relationship between ASD and the Scl Subscale but not with the Lng Subscale.

Figure 4 shows the graphical representation our hypothesized SEM model. The arrows between the latent factors represented within the circles are the relationships between the measures considered (ASD symptoms and the subscales Scl and Lng). Items are represented within rectangles, the arrows between factors (circles) and items are the item loadings. Each pathway has a label indicating its standardized estimate. The thickness of the paths indicates their strength.

The SEM model (Figure 4) with the measurement patterns of the considered measures shows a good fit to the data with the following fit indices: χ^2^(df = 50) = 62.542, *p* = 0.11; robust CFI = 0.95; robust RMSEA = 0.06, *p* = 0.34 (robust SRMR = 0.08). The latent constructs Social Interaction (SI), Communication and Behavior (CB), and Sensory/Stereotyped Behaviors (SSB) loaded significantly on a second‐order ASD factor, with CB showing the strongest contribution. Table 4 shows the item loadings on their latent factors and their explained variance (R^2^). In addition, the two GMDS subscales and ASD symptomatology show a significant relationship between ASD and the Scl Subscale (β = −0.023, SE = 0.10, *p* = 0.019), but not with the Lng Subscale (β = 0.01, SE = 0.01, *p* = 0.33) (Table 5).

**Table 4:** Item loadings and Explained Variance (R²) for First‐Order Latent Factors and Second‐Order Factor.

Items	Latent factor			
	SI	CB	SSB	R^2^
A2	1.00	—	—	0.349
B13	1.68	—	—	0.66
B14	1.14	—	—	0.38
B1	—	1.00	—	0.42
B4	—	1.42	—	0.59
B5	—	1.79	—	0.78
B6	—	1.58	—	0.46
B15	—	1.75	—	0.76
D1	—	—	1.00	0.60
D5	—	—	0.80	0.45

Factor	Construct			
	ASD	Personal‐Social	Language	R^2^
SI	1.00	—	—	0.73
CB	1.04	—	—	0.79
SSB	0.74	—	—	0.17

**Table 5:** Regression among ASD symptoms, Personal‐Social and Language subscale of GMDS.

Factor	Estimate	SE	z‐value	p
ASD∼	—	—	—	—
Social	−0.02	0.01	− 2.35	**0.019**
Language	0.01	0.01	0.95	0.33

*Note:* significant results are reported in bold

## 4 Discussion

The importance of multidisciplinary approaches, incorporating psychometric and analytical methods alongside clinical practice and diagnostic tools, is becoming central, particularly for understanding and attempting to standardise the symptomatic manifestations of ASD. Such approaches play a vital role in diagnosing and identifying children at risk early on. There is no evidence to suggest that the early manifestation of ASD symptoms is homogeneous enough to avoid being conceptualised through three symptom onset models: the early‐onset model, the regression model, and the ‘plateau’ model (Ozonoff and Iosif 2019; Shumway et al. 2011). To enhance efficiency and effectiveness in ASD assessment, ADOS‐2 has been examined in prior research to explore relationships between clinical evidence and attributes aligned with DSM‐5 criteria (Pino et al. 2018). In this study, EGA was applied to replicate and investigate the underlying structure of the Toddler Module items in an Italian sample of children aged 12 to 30 months. This analysis enabled us to compare EGA results with the structured task analysis, examining whether identified item clusters align with the behavioural categories assessed by the Toddler Module tests.

The EGA results indicated that the 14 items from the selected algorithm (‘All younger/older with few or no words’) generated 3 dimensions as the optimal structural solution. However, three items—A3 ‘Intonation of vocalisations and verbalisations’, B12 ‘Showing’, and D2 ‘Hand and finger movements/posture’—were inconsistent within the dimensions, compromising the overall network stability (Figure 2). Specifically, items A3 and D2, which theoretically belong to the RRB dimension (Lord et al. 2012), appeared unstable in our analysis, showing up in different dimensions from those hypothesised. This inconsistency may relate to the developmental characteristics of children in this age group. For example, item A3 may provide limited information about the investigated characteristics, especially for younger children or those with minimal verbal output. The ADOS‐2 manual suggests coding for ‘…*repetitive vocalisations or unusual nonwords’*, but this may not apply if the child does not vocalise sufficiently during assessment. Additionally, coding of A3 allows for an ‘8’ (not applicable), which converts to ‘0’ in the final score, representing a lack of information rather than an observed characteristic.

Item D2, on the other hand, codes for ‘*…unusual and repetitive movements or posture of hands and fingers’*. Its inconsistency with the dimensions may stem from its specificity because it focuses solely on hand movements, which may not be clearly evident during assessment. More complex movements, such as hand‐flapping, are instead coded in item D3 (‘*Other complex mannerisms’*), which is excluded from the final scoring algorithm. To achieve the best structural solution, we replicated the analysis, excluding inconsistent items and confirming a three‐dimensional solution with improved stability (Figure 3). Each item showed good to excellent stability and replicability indices, as well as robust network loadings (Table 3).

Therefore, from the analysis of the semantic content of the items grouped into the three dimensions identified by the EGA, these dimensions appear to represent domains related to Social Intentionality (Dimension 1), Communicative Behaviours (Dimension 2), and Sensory and Stereotyped Behaviours (Dimension 3). The EGA results for the Toddler Module provide insights into how the items and latent dimensions interrelate and how they are graphically positioned, highlighting the connections between latent dimensions (Christensen and Golino 2021).

Our results did not replicate the two‐factor solution of ADOS‐2 (Lord et al. 2012); however, the previous ADOS version was constructed to examine three dimensions, consistent with the DSM‐IV, which proposed a triadic model for diagnosing ASD. This triadic model included impairments in social interaction, communication, and the presence of restricted and repetitive behaviours and interests. With the update of DSM‐5 (American Psychiatric Association, D. S. M. T. F., & American Psychiatric Association, D. S 2013) and the adoption of a dyadic model, the two domains related to social interaction and communication were combined into a single dimension (i.e., Social Affect, or SA) in ADOS‐2.

Compared to the dyadic model used in the current version of ADOS‐2 (SA and RRB), our results therefore propose a model in which the SA area is divided into two distinct but interdependent dimensions, namely Social Intentionality and Communicative Behaviours. Furthermore, the items are grouped differently from the model currently in use.

In the Social Intentionality (SI) dimension, items A2, B14, and B15—which investigate behaviours reflecting ‘awareness’ of others and social reciprocity, skills that emerge with joint attention around 12 months of age (Happé and Frith 2014)—are grouped together. According to the ADOS‐2 manual, these items are coded based on the child's attempts to call for and respond to the caregiver's attention during assessment. For the Communicative Behaviours (CB) dimension, our analysis grouped items describing the child's communication methods within social interactions, encompassing the use of gestures, eye contact, and facial expressions, as well as behaviours indicating shared enjoyment and initiating social interaction with the caregiver. This separation suggests a more nuanced interpretation of early socio‐communicative development, which could align with the milestones in the development of the skills underlying social cognition processes, i.e., the set of skills that enable individuals to understand, interpret, and respond to the behaviours and emotions of others (Brothers 1990; Happé et al. 2017). It includes a continuum of skills ranging from basic abilities, such as face processing and biological motion perception, to more complex cognitive processes, such as the development of joint attention and the ability to understand and interpret one's own and others’ mental states, i.e. Theory of Mind. The development of these skills occurs along a continuum, following specific stages, until they become a complex network of functions, or “network”, consisting of processes, or “nodes”, which in turn branch out into sub‐processes, all of which are interdependent and interconnected (Happé and Frith 2014). It is precisely the impairment of the development of these skills that is now widely considered to be the core deficit in ASD (Baron‐Cohen et al. 2001; Mazza et al. 2017). The model obtained from our analyses would allow us to identify more specifically any impairment of basic skills for adequate social interaction. Finally, as proposed in ADOS‐2, items assessing sensory interests and repetitive behaviours (D1 and D5) remain in the hypothesised dimension. In our sample of children at risk for ASD, however, sensory interests and stereotypic behaviours appear to hold particular significance. Therefore, while the RRB domain remains consistent with the ADOS‐2 framework, with the exception of the removal of two items considered inconsistent with the dimension, the subdivision of the social domain can provide information on specific profiles or trajectories within the autism spectrum, particularly in younger children.

The model proposed by the results obtained from the EGA could be influenced by the methodology itself. Unlike traditional factorial techniques, the psychometric network approach identifies dimensions based on covariance models, without imposing a predefined theoretical structure (Baumert et al. 2017), making it more sensitive to the identification of dimensions (Golino et al. 2020).

So, based on the EGA results, the dimensional structure was also tested using a CFA model, which demonstrated good fit indices. To move from construct and dimensionality analysis to applying the structured tasks of the instrument, we compared the aggregation of items within the dimensions derived from the Toddler Module tests. It appears that four tasks elicit the main features represented by the three dimensions (SI, CB, and SSB): ‘Free Play’, ‘Bubble Play’, ‘Anticipating a Routine with Objects’, and ‘Bath Time’ (see Supplementary Materials). This suggests a structure of ‘main’ tasks and ‘optional/complementary’ tasks for administering these structured tasks. ‘Free Play’ would represent the main task, in which a situation is created where the child is not prompted by the examiner. This task allows for the assessment of the child's engagement with the caregiver (or examiner), communication and affection patterns, and presence of stereotypical behaviours. Within this context, the examiner may also assess specific areas, such as name response, social smiling, and joint attention response.

The ‘Bubble Play’ task and ‘Anticipating a Routine with Objects’ task, along with their subtests (‘Joke’ and ‘Play No More’), allow for coding of the same items and features—namely, engagement, shared enjoyment, spontaneous attention initiation, repetitive behaviours, and reactions to unusual social situations created by the examiner. If the examiner can code these items through, for example, ‘Bubble Play’, it may be redundant to administer an additional task that would elicit the same characteristics. Instead, these tasks could complement each other when it is necessary to investigate the same characteristic (or item) in multiple contexts. During assessment, it is not unusual to observe adverse reactions in the ‘Anticipating a Routine with Objects’ task (e.g., the child experiences fear from the noise of the inflated balloon) or a lack of interest, which may lead to discontinuation of the task. Similar reactions may also occur in ‘Bubble Play’. Such a structured approach could have significant implications for clinical practice, as it would enable efficient information gathering for diagnosis, reducing administration time, and minimising the need for prolonged cooperation from the child. This is particularly beneficial in assessments of very young children, who often demonstrate characteristics of this early developmental stage, such as test reactivity (e.g., tiring easily) and limited attentional capacity. Similar results were achieved using innovative analytical methods, such as FPA (Pino et al. 2018), which enabled analysis of the relationships between items across all five modules of ADOS‐2 and the clinical attributes for ASD diagnosis provided by DSM‐5 (American Psychiatric Association, D. S. M. T. F., & American Psychiatric Association, D. S 2013). This analysis identified prerequisite relationships between different tasks in the Toddler Module, such as ‘Bubble Play’ preceding the ‘Anticipating a Routine with Objects’ task or ‘Functional and Symbolic Imitation’ preceding the ‘Bath Time’ administration. Thus, making the assessment more adaptive would allow for reduced administration time and a focus on specific diagnostic aspects without repeatedly investigating the same characteristic (Pino et al. 2018). Regarding the second objective of the study, we developed a theoretical model to contribute to the characterisation of ASD symptomatology and explore the integration of measures that investigate specific features of the condition. It is well established that ASD is a neurodevelopmental disorder, and early developmental indicators play a crucial role in gathering information to support and confirm an early diagnosis. Previous evidence has shown that social behaviour indicators are associated with the early onset of ASD symptoms and symptom severity at the time of diagnosis (Ozonoff and Iosif 2019; Pino et al. 2024). In our SEM model, we examined the relationships between clinical constructs, specifically ASD symptomatology—conceptualised with the three dimensions identified in the EGA results—and cognitive development in social and communication skills, represented by the two GMDS subscales selected based on prior evidence (Personal‐Social and Language).

The results of the SEM model (Figure 4) show discrete fit indices and indicate how the grouped items define the SI, CB, and SSB dimensions, which are associated with overall ASD symptomatology. The model also reveals a significant influence of social behaviour development skills on ASD symptomatology, consistent with previous evidence. Contrary to our expectations, the Language subscale—an indicator of communicative development—does not appear to be a significant predictor of ASD symptomatology. This may be because the communicative development assessed by the GMDS reflects the acquisition of receptive and expressive components, such as repeating words or short sentences, naming objects and colours, or answering simple questions. Although communicative development might be impaired in the ASD population, it has been shown to increase significantly from infancy to elementary school (Thomas et al. 2021). What remains impaired are the semantic and pragmatic features, which become evident when the child with ASD attempts social interaction.

Smiling and following people with the gaze are examples of items assessed by the Personal‐Social subscale and represent some of the skills that infants are predisposed to develop early (Happè & Frith, 2014). In children with ASD, however, there is often an alteration in the timing of acquisition for these precursors to more complex social skills.

These findings further support previous evidence that early signs of altered social behaviour are present within the first year of life, although identification may be challenging. The results from the present study provide valuable support for clinical practice, encouraging the use and integration of selected instruments during early assessment.

## 5 Limitations

Some limitations of the present study should be acknowledged. The two samples of children were small, particularly in the second study, which represents a limitation that future studies could address to replicate these results and improve the SEM model's fit indices; specifically, the RMSEA value is likely nonsignificant due to limited degrees of freedom. Another limitation related to the sample is the absence of an independent sample, which limits the generalizability of our findings and increases the risk of overfitting, particularly in structural equation modeling. However, we would like to point out that the SEM model presented is exploratory and preliminary in nature, with the aim of testing the consistency of the structure identified through EGA. The results of this second phase should be interpreted with caution and confirmed in future studies conducted on independent and larger samples, with a view to external validation.

Another limitation is that we analysed, albeit preliminarily, only one diagnostic algorithm of the Toddler Module (‘All younger/older with few or no words’) because the number of children assessed with the module's second algorithm (‘Older with some words’) was very small.

Furthermore, the “adaptive” structure, based on “main” and “complementary/optional” tasks, represents a conceptual proposal developed from the results obtained using the EGA psychometric model and from post hoc observations on the association between items and tasks within the Toddler Module. Since this proposal has not been empirically tested, it should be considered preliminary and worthy of specific investigation in future studies that systematically evaluate its clinical feasibility and diagnostic validity.

## 6 Conclusions

We believe that our results have relevance not only for research on the characterisation of ASD symptomatology but also for clinical and rehabilitation practice. Making early assessment more efficient and effective by selecting ‘main’ and ‘optional/complementary’ tests has implications not only for reducing administration time but also for optimising information collection. Additionally, integrating measures to place greater emphasis on key characteristics and indicators for identifying children at risk for ASD—while deferring other components to later assessment—could facilitate timely, targeted interventions, ultimately improving the prognosis. However, it is important to emphasize that the proposed adaptive structure, as well as the three‐factor model that emerged from our exploratory analysis, should be considered preliminary and hypothesis‐generating. This model can offer a more detailed perspective on early socio‐communicative and behavioral profiles and highlights potential directions for future research aimed at validating these findings in larger, independent samples and assessing their clinical utility in different developmental contexts.

## Author Contributions

Ilenia Le Donne and Monica Mazza designed the research and the methodology, collected and analyzed the data, writing the original draft. Margherita Attanasio contributed to the analysis and interpretation of data, reviewed the manuscript. Nicole Covone, Maria Paola Greco and Veronica Scurti contributed equally to collecting and preparation data. Marco Valenti contributed to conceptualization of the study and the critical revision and supervision of the work. All authors read and edited the manuscript and approved the final version.

## Ethics Statement

The study was conducted under the Declaration of Helsinki and the rules of good clinical practice.

## Consent

The Ethics Committee of the NHS Local Health Unit 1 (Azienda Sanitaria Locale 1; ASL 1—Avezzano, Sulmona, L'Aquila), Abruzzo Region, L'Aquila, Italy, approved the experimental protocol (n. 0052505/21).

## Conflicts of Interest

The authors declare no conflicts of interest.

## Supporting information


**Table SM1:** Correspondence of the structured tests of the Toddler Module, with the respective items and latent dimensions emerged from the EGA model.

## Data Availability

The data that support the findings of this study are available from the corresponding author upon reasonable request.
