# Supplementary material for: Application of Psychometric Methods in Dimensional Analysis and Integration of Assessment Tools in Early Diagnosis for Autism Spectrum Disorder
Source: J Clin Psychol. 2025 Oct 26;82(3):260–72. doi: 10.1002/jclp.70059 (PMC12882800; doi:10.1002/jclp.70059)
Supplement: Supplementary file 1 — Table SM1: Correspondence of the structured tests of the Toddler Module, with the respective items and latent dimensions emerged from the EGA model. [file JCLP-82-260-s001.docx]

**Supplementary Material**

**Table SM1.** Correspondence of the structured tests of the Toddler Module, with the respective items and latent dimensions emerged from the EGA model.

| **Task** | **Item** | **Dimension** |
| --- | --- | --- |
| 1. **Free Play**   **1a. Free Play-Ball** | A2. Frequency of spontaneous vocalizations directed at others  A3. Intonation of vocalizations and verbalizations  B1. Unusual eye contact  B4. Facial expressions directed to others  B6. Shared enjoyment in interaction  B13. Spontaneous initiation of joint attention  B12. Showing  B15. Quality of social initiations  D1. Unusual sensory interest in play materials/people  D2. Hand and finger movements/posture  D5. Unusual repetitive interests or stereotypical behaviors | SI  CB  SSB |
| 1. **Block access to the game** | A2. Frequency of spontaneous vocalizations directed at others  B1. Unusual eye contact  B4. Facial expressions directed to others | SI  CB |
| 1. **Response to the name** | A2. Frequency of spontaneous vocalizations directed at others  B1. Unusual eye contact  B4. Facial expressions directed to others | SI  CB |
| 1. **Bubble Game**   **4a. Joke** | A2. Frequency of spontaneous vocalizations directed at others  A8. Gestures  B1. Unusual eye contact  B4. Facial expressions directed to others  B5 Integration of gaze and other behaviors during social initiations  B6 Shared enjoyment in the interaction  B13 Spontaneous initiation of joint attention  B14 Joint attention response  B15 Quality of social initiations  D1. Unusual sensory interest in play materials/people  D2. Hand and finger movements/posture  D5. Unusual repetitive interests or stereotypical behaviors | SI  CB  SSB |
| 1. **Anticipating A Routine With Objects**   **5a. Play No More** | A2. Frequency of spontaneous vocalizations directed at others  A8. Gestures  B1. Unusual eye contact  B4. Facial expressions directed to others  B5 Integration of gaze and other behaviors during social initiations  B6 Shared enjoyment in the interaction  B13 Spontaneous initiation of joint attention  B14 Joint attention response  B15 Quality of social initiations  D1. Unusual sensory interest in play materials/people  D2. Hand and finger movements/posture  D5. Unusual repetitive interests or stereotypical behaviors | SI  CB  SSB |
| 1. **Anticipation Of Social Routine** | A2. Frequency of spontaneous vocalizations directed to others  A8. Gestures  B1. Unusual eye contact  B4. Facial expressions directed to others  B5. Integration of gaze and other behaviors during social initiations  B6. Shared enjoyment in the interaction  B13. Spontaneous initiation of joint attention  B15. Quality of social initiations | SI  CB |
| 1. **Joint Attention Response** | B1. Unusual eye contact  B14. Joint attention response | SI  CB |
| 1. **Social Smile Response** | A2. Frequency of spontaneous vocalizations directed to others  B1. Unusual eye contact  B4. Facial expressions directed to others  B5. Integration of gaze and other behaviors during social initiations  B6. Shared enjoyment in the interaction | SI  CB |
| 1. **Baby Bath**   **9a. Ignore** | A2. Frequency of spontaneous vocalizations directed to others  B1. Unusual eye contact  B4. Facial expressions directed to others  B6. Shared enjoyment in the interaction  B13. Spontaneous initiation of joint attention  B15. Quality of social initiations  D2. Hand and finger movements/posture  D5. Unusual repetitive interests or stereotypical behaviors | SI  CB  SSB |
| 1. **Functional And Symbolic Imitation** | B6. Shared enjoyment in the interaction | CB |
| 1. **Snack** | A2. Frequency of spontaneous vocalizations directed to others  A8. Gestures  B1. Unusual eye contact  B4. Facial expressions directed to others  B5. Integration of gaze and other behaviors during social initiations  B15. Quality of social initiations | CB |

**Note.** Items that were found to be unstable through EGA analysis and eliminated from the dimensions are shown in red; SI: Social Intentionality; CB: Communicative Behaviors; SSB: Sensory and Stereotyped Behaviors.
